# A retrospective study (2007–2015) on brucellosis seropositivity in livestock in South Africa

**DOI:** 10.1002/vms3.363

**Published:** 2020-10-22

**Authors:** Francis B. Kolo, Abiodun A. Adesiyun, Folorunso O. Fasina, Andrew Potts, Banenat B. Dogonyaro, Charles T. Katsande, Henriette Van Heerden

**Affiliations:** ^1^ Department of Veterinary Tropical Diseases University of Pretoria Pretoria South Africa; ^2^ Department of Production Animal Studies University of Pretoria Pretoria South Africa; ^3^ Onderstepoort Veterinary Research Pretoria South Africa; ^4^ Gauteng Department of Agriculture and Rural Development Johannesburg South Africa

## Abstract

In South Africa, brucellosis testing and record‐keeping are done by several laboratories, thus it is difficult to access any organized data to assess the status of the disease. This study evaluated the seropositivity for brucellosis using Rose Bengal test and complement fixation test in suspect cattle, sheep, goats and pigs sera submitted to Bacterial Serology Laboratory, Agricultural Research Council‐Onderstepoort Veterinary Research (ARC‐OVR) from nine provinces in the country during the period 2007–2015. This retrospective data analysis was conducted to estimate the occurrence of brucellosis in the country from the submitted samples, identify variables that affected seropositivity for brucellosis, investigate existing gaps in data recording and make recommendations on important variables to facilitate better data capture and inferences on brucellosis. Nine years of data were collated and analysed to detect association (seropositivity over time regarding animal species and location). Of the 764,276 animals tested, the distribution of samples was 90.50% (691,539/764,276), 5.19% (39,672/764,276), 3.92% (29,967/764,276) and 0.41% (3,098/764,276) for cattle, sheep, goats and pigs, respectively. The seropositivity for brucellosis by animal species was 6.31% (43,666/691,539, 95% CI: 6.26–6.37), 2.09% (828/39,672, 95% CI: 1.95–2.23), 0.63% (189/29,967, 95% CI: 0.55–0.73) and 0.13% (4/3,098, 95% CI: 0.05–0.33) in cattle, sheep, goats and pigs respectively. The data available did not capture information on the age, sex, breed and other host risk factors that would have been related to seropositivity for brucellosis. The data provide an understanding of the disease occurrence and confirm that brucellosis is enzootic in South Africa. Improved and standardized data collection can be used to pro‐actively drive, monitor, change or formulate policies to mitigate the challenges brought about by brucellosis in the livestock sector in South Africa.

## Graphical abstract

A retrospective study on brucellosis in South Africa.

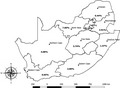

## 1 INTRODUCTION

Brucellosis is one of the most important and widespread zoonoses in the world (Kolar et al., 1995). Twelve *Brucella* species have been isolated of which *B. abortus, B. melitensis* and *B. suis* have been reported to affect livestock and humans while *B. ovis* affects only livestock (El‐Sayed & Awad, 2018). In livestock, brucellosis is characterized by abortions and reproductive failure (Corbel & World Health Organization, 2006) but after abortion, the females can give birth again but continue to shed the pathogen (Lopes et al., 2010). Infections in humans cause additional losses (financial and disease burden), with prolonged clinical symptoms which could vary from months to years (Abdou, 2000; Corbel & World Health Organization, 2006; Zajtchuk & Bellamy, 1997). Brucellosis is an occupational disease and poses a risk to abattoir workers, employees in the meat packaging industry, veterinarians and farmers (Corbel & World Health Organization, 2006; Young, 1995).

In developed countries brucellosis is well controlled (Pappas et al., 2006) through routine domestic livestock surveillance, screening and animal vaccination programmes (Corbel, 1997; Maloney & Fraser, 2001). Brucellosis remains common in Africa, south and Central America, the Middle East, Asia, the Mediterranean basin and the Caribbean (Pappas et al., 2006). Brucellosis in the animal and human populations in South Africa date back to the early 19th century in various parts including Philippolis (Free State province), Steytlerville (Eastern Cape province) and Northern Cape province districts (Strachan, 1932; Van Drimmelen, 1949). In South Africa *B. abortus* infection was documented in 1913, when contagious abortion was observed to spread in cattle across the country and cases of ‘camp fever’ documented in humans (Thornton, 1936; Van Drimmelen, 1949). The fact that human cases of brucellosis were documented more than a century ago in South Africa could indicate that the disease had been circulating in the animal population. In the 1920s, only *B. melitensis* was isolated and suspected to be the cause of ‘camp fever’ or Malta fever in South Africa. Prior to these cases, it had been suspected in 1898 that goats may have been the source of suspected cases of ‘camp fever’ in 40 human patients around the Kimberley area (Northern Cape province) of South Africa (Strachan, 1932; Van Drimmelen, 1949). Brucellosis diagnosis had always been conducted with serological tests and bacteriological isolation (*B. melitensis*) from 1902 to 1911 (Strachan, 1932; Zammit, 1905), and these tests have yielded results from human blood (*B. abortus*) as well as from goat serum and milk samples in South Africa (Strachan, 1932). Later from 1956 to 1959 *B. abortus* and *B. melitensis* were isolated from human blood samples as well (Schrire, 1962).

In South Africa brucellosis is a reportable and priority disease. The control scheme is focused primarily to prevent the spread of bovine brucellosis and involves vaccination with *B. abortus* S19 and *B. abortus* RB 51 in cattle and *B. melitensis* Rev 1 in sheep, test and slaughter as well as prohibition of the movement of live animals from infected herds other than those for slaughter (OIE, 2016). The *B. abortus* S19 vaccine is used at the government recommended dose of 5 × 10^10^ organisms on 4–8‐month‐old heifers (DAFF, 2016). Testing of animals for brucellosis is voluntary, except for dairy cattle where serological testing using Rose Bengal test (RBT) and complement fixation test (CFT) is compulsory. The South African government is currently funding brucellosis serological tests and culture, if samples are submitted through State Veterinary Services to veterinary laboratories (consisting of provincial laboratories and the Bacterial Serology Laboratory, ARC‐OVR). The brucellosis test results from Veterinary laboratories are not centralized and conducted currently by the Bacterial Serology Laboratory, ARC‐OVR in Gauteng province and a few provincial laboratories (North West, KwaZulu‐Natal and Western Cape provinces) that each keep separate records. The estimated average number of livestock by province in South Africa during the study period is shown in Table S1.

The goal of this study was to estimate the occurrence of brucellosis in the country from the submitted livestock samples, identify variables that had significant effects on seropositivity for brucellosis, investigate existing gaps in data recording and recommend on important variables to facilitate better data capture and inferences on brucellosis. The study design was also to relate *Brucella* serology to time and species for a 9‐year period (2007 to 2015) from all the nine provinces of South Africa, on data relating to samples received at the Bacterial Serology Laboratory, ARC‐OVR.

## 2 MATERIALS AND METHODS

### 2.1 Study design

The study design was to acquire and collate diagnostic brucellosis data from samples collected from suspected cases of animal brucellosis tested at the Bacterial Serology Laboratory, ARC‐OVR. Data abstraction was conducted by identifying complete and useable variables among available data set. Useable variables were filtered and curetted in Microsoft Excel 2007 version, and were stratified according to the year of testing, animal species, province and then related to the outcome of the serial testing conducted on the samples.

### 2.2 Study area

South Africa is in the tropic of Capricorn in the southern hemisphere and the southernmost tip of the continent of Africa. The human population is estimated to be 58 million people with a surface area of 1,219,602 km^2^. The country has several distinct ecosystems and it is bounded by 2,798 km of coastline stretching along the South Atlantic and the Indian Oceans. In the north, its neighbouring countries are Namibia, Botswana, Zimbabwe and to the east and northwest are Mozambique and Swaziland. The country is divided into nine provinces and hosting the provincial laboratories and the Bacterial Serology Laboratory, ARC‐OVR, which is in Gauteng province. The distribution of the number animals in the provinces (DAFF, 2018) during the study period is shown in Table S1.

The South African government announced that veterinary laboratories must be accredited to conduct the brucellosis serological testing from 2010. The Bacterial Serology Laboratory, ARC‐OVR was the first veterinary laboratory to be accredited to conduct brucellosis serological tests in 2010 and overtime provincial veterinary laboratories were accredited. Currently the four provincial veterinary laboratories that are approved by the South African government (DAFF, 2020) to conduct serological tests for brucellosis are: Allerton Provincial Veterinary Laboratory in KwaZulu‐Natal, Northern Cape Provincial Veterinary Laboratory, Potchefstroom Provincial Veterinary Laboratory in North West, Western Cape Provincial Veterinary Laboratory. The three approved private laboratories include: Pathcare N1 Veterinary Laboratory in Cape Town (Western Cape), Capricorn Veterinary Laboratory in Polokwane (Limpopo) and Veterinary Tropical Diseases Serology, University of Pretoria (Gauteng).

### 2.3 Sampling

Records from 2007 to 2015 were retrieved from the Bacterial Serology Laboratory, ARC‐OVR in South Africa. These data consisted of serological results of tests conducted on animal samples (serum) sent from farms, veterinary clinics, regional provincial laboratories and from the animal health officers from the nine provinces. Suspicion of brucellosis was the primary criterion for the samples sent to the laboratory for testing and confirmation testing on the same animal could not be differentiated. Other samples sent for testing for brucellosis included animals from farms to confirm their brucellosis‐free status and those destined to be exported to other countries.

### 2.4 Laboratory tests data

Serial testing programme method was used to analyse all sera at ARC‐OVR Laboratory (i.e. RBT positive followed by CFT). The RBT was used as a screening test for individual animals (Alton et al., 1988; OIE, 2009), while the CFT was used to confirm brucellosis in RBT‐positive samples (Alton et al., 1988; OIE, 2009). Seropositivity for brucellosis was estimated based on samples seropositive on both RBT and CFT in series, and with titres of ≥1:30 for CFT. The correlation of the bacteriological and serological test results could not be investigated primarily because the two tests (serology and bacteriology) are conducted by different departments at ARC‐OVR.

### 2.5 Statistical analysis

The data were collated and managed in Microsoft Excel 2007 version and descriptive analysis was conducted using R (R Core Team, 2013). The data were analysed based on the frequency of brucellosis seropositivity stratified by livestock species, province and year of testing. Analyses of measures of association of *Brucella* seropositivity with plausible risk factors and predictors (livestock species, year and provinces) were conducted using the Two X Two Table in OpenEpi^®^ (https://www.openepi.com/TwobyTwo/TwobyTwo.htm). For this purpose, the year ‘2007’, ‘KwaZulu‐Natal Province’ and ‘pigs’ were used as reference for comparison of risk within the categories.

## 3 RESULTS

### 3.1 Demographic details

**FIGURE 1:**
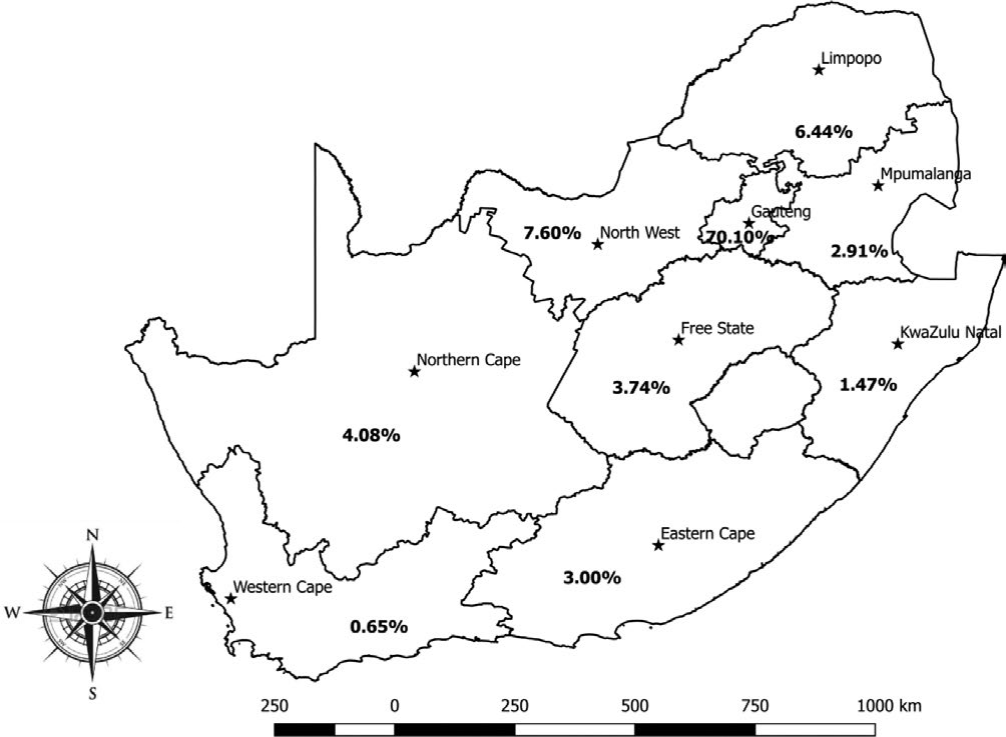
Sources of animal samples submitted for brucellosis testing (2007–2015) shown by province of origin in South Africa

In our study we simply abstracted data available at the ARC‐OVR Laboratory and no serological tests were conducted in the current study. The current study conducted data retrieval, filtration, curation and closely assessed the data to identify all the variables available for each serum sample: animal species, province, year of test, individual sender, post code, farm sources and coordinates. Considering that information on individual sender, post code, farm sources and coordinates were incomplete or unavailable; the only data available that could be subjected to statistical analysis were animal species and location. Of the 764,276 animals tested from 2007 to 2015, the largest proportion was cattle with 90.50% (691,539/764,276), while 5.19% (39,672/764,276), 3.92% (29,967/764,276) and 0.40% (3,098/764,276) were sheep, goats and pigs, respectively. The distribution based on the provinces ranged from 0.65% (4,941/764,276) in Western Cape to 70.10% (535,762/764,276) in Gauteng where ARC‐OVR is located (Figure 1). The distribution of samples tested for the other provinces is as follows; 22,927, 11,239, 49,248, 22,267, 31,220, 58,099 and 28,572 for Eastern Cape, KwaZulu‐Natal, Limpopo, Mpumalanga, Northern Cape, North West and Free State respectively (Figure 1).

### 3.2 Seropositivity and analysis of risk factors

**FIGURE 2:**
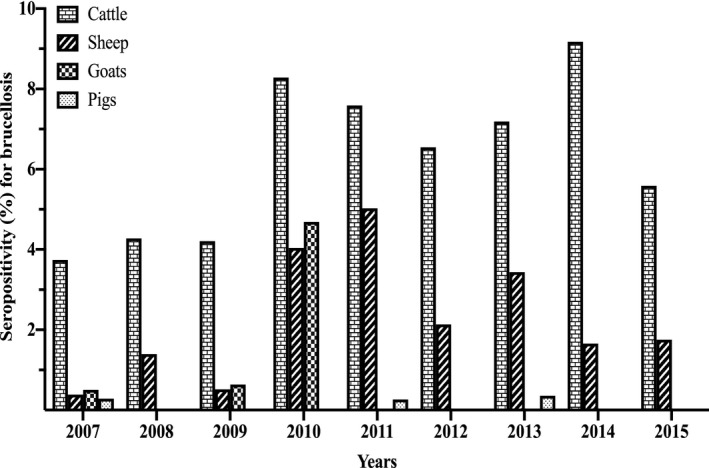
Seropositivity for brucellosis in livestock in South Africa from 2007–2015 recorded data by year at the Bacterial Serology Laboratory, ARC‐OVR

**FIGURE 3:**
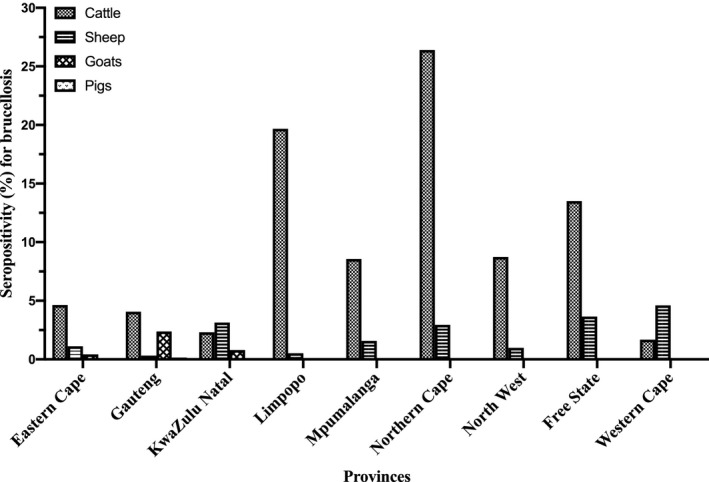
Seropositivity for brucellosis in livestock in South Africa by province from 2007–2015 recorded data at the Bacterial Serology Laboratory, ARC‐OVR

The seropositivity for brucellosis stratified by animal species for the period 2007 to 2015 (Figure 2; Table S2) shows that the seropositivity for brucellosis in cattle varied from 3.74% (2007) to 9.18% (2014), in goats from 0.00% (2008, 2013, 2014) to 4.69% (2010), in sheep from 0.39% (2007) to 5.03% (2011) and in pigs from 0.00% (2008, 2009, 2010, 2012, 2014, 2015) to 0.36% (2013). Overall, for all animal species tested in the 9‐year period, the seropositivity for brucellosis was 5.85% (44,687/764,276, 95% CI: 5.79–5.90). The seropositivity for brucellosis by animal species for the study period was 6.31% (43,666/691,539, 95% CI: 6.26–6.37), 2.09% (828/39,672, 95% CI: 1.95–2.23), 0.63% (189/29,967, 95% CI: 0.55–0.73) and 0.13% (4/3,098, 95% CI: 0.05–0.33) in cattle, sheep, goats and pigs, respectively (Figure 3, Table S3). Of the brucellosis seropositive animals (*n* = 44,687), cattle had the highest occurrence by proportion (*n* = 43,666; 97.72%, 95% CI: 97.57–97.85), followed by sheep (*n* = 828; 1.85%, 95% CI: 1.73–1.98), goats (*n* = 189; 0.42%, 95% CI: 0.37–0.49) and pigs with the lowest proportion of positives (*n* = 4; 0.01%, 95% CI: 0.00–0.02). For the provinces, there was a wide disparities in the distribution of seropositive animals as shown in Table S3 and Figure 3. Briefly, Limpopo province had the highest overall seropositive rate of 17.65% (8,695/49,248) followed by Northern Cape province (16.80%, 5,259/31,220) with the lowest being Western Cape province (1.84%, 91/4,941) (Figure 3, Table S3). Species discrimination in levels of seropositivity exists within the Provinces.

For the measure of association of risk factors and predictors, using the year 2007 as a reference, the highest odds ratios for seropositivity to *Brucella* antibodies occurred as from the year 2010 for all animal species. For instance, the year 2014 was 2.6 times more likely to present with *Brucella* seropositive animals (χ^2^ = 1749, *p* < .0001) for cattle; the year 2010 was 9.6 times more likely to present with *Brucella* seropositive animals (χ^2^ = 114, *p* < .0001) for goats; the year 2011 was 13.4 times more likely to present with *Brucella* seropositive animals (χ^2^ = 195.1, *p* < .0001) for sheep; and, the year 2013 was 1.3 times more likely to present with *Brucella* seropositive animals (χ^2^ = 0.03, *p* = .85) for pigs. Similar observations were observed for provinces. Cattle is 52 times more likely to be seropositive compared with pigs (χ^2^ = 200.3, *p* < .0001), while sheep and goats are more likely to be 16.5 (χ^2^ = 57.8, *p* < .0001) and 5(χ^2^ = 12.2, *p* = .0005) times seropositive compared with pigs (Table 1).

**TABLE 1:** Risk factors and predictors for seropositivity from 2007 to 2015 retrospective data and stratified by year, provinces and species in South Africa

*By year*	Cattle tested (^a^n = 691,539)			Goats tested (^a^n = 29,967)			Sheep tested (^a^n = 39,672)			Pigs tested (^a^n = 3,098)		
	^b^OR	Chi square	*p*‐value	^b^OR	Chi square	*p*‐value	^b^OR	Chi square	*p*‐value	^b^OR	Chi square	*p*‐value
2007	^c^Ref	‐	^d^NA	^c^Ref	‐	^d^NA	^c^Ref	‐	^d^NA	^c^Ref	‐	^d^NA
2008	1.15 (1.09; 1.21)	25.87	<.0001	^d^NA	‐	^d^NA	3.59 (2.21; 6.16)	30.4	<.0001	^d^NA	‐	^d^NA
2009	1.13 (1.07; 1.19)	21.22	<.0001	1.26 (0.67; 2.37)	0.49	.48	1.33 (0.74; 2.39)	0.92	.34	^d^NA	‐	^d^NA
2010	2.33 (2.22; 2.44)	1,281	<.0001	9.58 (5.79; 15.87)	114	<.0001	10.64 (6.75; 16.79)	159.5	<.0001	^d^NA	‐	^d^NA
2011	2.11 (2.01; 2.22)	949.6	<.0001	0.05 (0.006; 0.36)	17.71	<.0001	13.39 (8.37; 21.40)	195.1	<.0001	0.92 (0.08; 10.17)	0.005	.95
2012	1.80 (1.71; 1.90)	538.1	<.0001	0.07 (0.009; 0.51)	12.01	.0005	5.53 (3.34; 9.18)	55.31	<.0001	NA	‐	^d^NA
2013	1.99 (1.90; 2.09)	829.2	<.0001	^d^NA	‐	^d^NA	9.01 (5.39; 15.05)	102	<.0001	1.26 (0.11; 13.91)	0.03	.85
2014	2.60 (2.48; 2.72)	1749	<.0001	^d^NA	‐	^d^NA	4.27 (2.60; 7.01)	39	<.0001	^d^NA	‐	^d^NA
2015	1.52 (1.45; 1.60)	285.9	<.0001	0.05 (0.007; 0.40)	16.18	<.0001	4.53 (2.77; 7.41)	43.5	<.0001	^d^NA	‐	^d^NA
*By province*												
KwaZulu Natal	^c^Ref	‐	^d^NA	^c^Ref	‐	^d^NA	^c^Ref	‐	^d^NA	^c^Ref	‐	^d^NA
Gauteng	1.79 (1.55; 2.08)	62.76	<.0001	3.06 (1.93; 4.85)	25.19	<.0001	0.09 (0.04; 0.21)	53.34	<.0001	^d^NA	‐	^d^NA
Eastern Cape	2.06 (1.73; 2.45)	69.67	<.0001	0.54 (0.28; 1.02)	3.73	.05	0.35 (0.20; 0.60)	15.59	<.0001	^d^NA	‐	^d^NA
Limpopo	10.38 (8.95; 12.03)	1,451	<.0001	^d^NA	‐	^d^NA	0.16 (0.07; 0.36)	26.72	<.0001	^d^NA	‐	^d^NA
Mpumalanga	3.97 (3.41; 4.63)	357.4	<.0001	^d^NA	‐	^d^NA	0.49 (0.23; 1.04)	3.56	.06	^d^NA	‐	^d^NA
Northern Cape	15.22 (13.11; 17.67)	2,108	<.0001	^d^NA	‐	^d^NA	0.93 (0.55; 1.59)	0.06	.8	^d^NA	‐	^d^NA
North West	4.06 (3.50; 4.71)	393.8	<.0001	0.06 (0.02; 0.18)	48.02	<.0001	0.30 (0.17; 0.55)	17.45	<.0001	^d^NA	‐	^d^NA
Free State	6.61 (5.68; 7.70)	765	<.0001	0.06 (0.008; 0.43)	14.59	.0001	1.17 (0.69; 1.97)	0.33	.56	^d^NA	‐	^d^NA
Western Cape	0.73 (0.55; 0.96)	5.22	.02	^d^NA	‐	^d^NA	1.49 (0.75; 2.94)	1.32	.25	^d^NA	‐	^d^NA
*By species*	^b^OR	Chi square	*p*‐value	Details of the absolute numbers and percentages of positive samples by year, provinces and species are available in the Supplementary Materials Tables S1, S2 and S3.								
Pigs	^c^Ref	‐	NA									
Cattle	52.13 (19.55; 139.0)	200.3	<.0001									
Goats	4.91 (2.01; 15.60)	12.17	.0005									
Sheep	16.49 (6.17; 44.06)	57.76	<.0001									

^a^ Number.

^b^ Odds ratio.

^c^ Reference.

^d^ Not applicable.

## 4 DISCUSSION

In South Africa, the existing surveillance system is weak in monitoring brucellosis in livestock on farms and/or abattoirs, or high‐risk human population or from diagnostic laboratories records (DAFF, 2017; Padilla et al., 2010). Although *Brucella* species have been isolated from livestock (Caine et al., 2017; Kolo et al., 2019; Van Drimmelen, 1949, 1965) and humans (Schrire, 1962; Wojno et al., 2016), much is needed to be done to strengthen surveillance. Low prevalence of brucellosis has been documented in previous studies in South Africa which include the 1.50% reported for cattle sampled at the Cato Ridge abattoir in Kwazulu‐Natal province in 1984 (Bishop, 1984), 1.45% in rural cattle sampled in communities in KwaZulu‐Natal from 2001 to 2003 (Hesterberg et al., 2008) and more recently, 5.50% seropositivity in slaughtered cattle at Gauteng province abattoirs (Kolo et al., 2019).

The Gauteng Department of Agriculture and Rural Development (GDARD) reported 1.27% (30/2,359) seroprevalence in the cattle population in Gauteng province from 2015 to 2016 using the RBT and CFT tests (GDARD, 2016). The testing was done by the ARC‐OVR and reflected in our results. The current study revealed an overall seropositivity of 5.85% (44,687/764,276) for brucellosis in livestock (cattle, sheep, pigs and goats), with individual animal species seropositivity at 6.31%, 2.09%, 0.63% and 0.13%, respectively from 2007 to 2015 at the Bacterial Serology Laboratory, ARC‐OVR. Retrospective review of seroprevalence observed and documented in Sub‐Saharan Africa in livestock (Ducrotoy et al., 2017) were 1.00%–10.60% in cattle in countries where two serological tests were used in series (same criteria used in our study) were comparable to the 6.31% we detected. The analysis in this study identified the primary focus on bovine brucellosis as a gap in recording findings. Since the brucellosis scheme in South Africa mainly focuses on bovine, it was no surprise that the over‐whelming majority of livestock tested was cattle, 90.50% (691,539/764,276).

The bovine brucellosis scheme is historic and based on the anecdotal belief that brucellosis is mainly a problem in cattle in the country. Since the brucellosis scheme in South Africa is biased towards the cattle population while sheep, goats and pigs have not received much attention, this could be mis‐interpreted as though a higher seropositivity of brucellosis was detected in cattle compared to sheep, goats and pigs. The focus on bovine brucellosis could also be that cattle are considered economically productive based on meat and milk production as well as their export potential in South Africa compared to sheep, goats and pigs. It is relevant to mention that a limited level of control measures observed for cattle is being applied to some sheep and goat population in the country as *B. melitensis* outbreaks mainly identified with human brucellosis cases has been controlled in associated goat and sheep populations (Emslie & Nel, 2002; Kolar, 1987). The serological data of goats from ARC‐OVR with 4.69% seropositivity in 2010 compared to 0.00%–0.64% in other years (Figure 2) reflect the reported *B. melitensis* outbreak in goats in Gauteng province in that year (DAFF, 2015).

The centralization of brucellosis data from veterinary laboratories would provide a more extensive picture of brucellosis in South Africa. The centralized data will still reflect the level of brucellosis detection in high‐risk cattle herds (with suspect cases of the disease) and not the level in most/all cattle in South Africa. Data from sheep and goats will be even more biased as there is no routine testing for these species. In South Africa, there is very little attention paid to the control of porcine brucellosis, primarily because the disease has not been reported in pigs in the country. This may be the reason why vaccination as control measures for brucellosis in pigs is not practised in the country. Testing for porcine brucellosis is complex because serological cross‐reactions have been documented between *Brucella* spp. and *Yersinia enterocolitica* serotype O:9 (Gerbier et al., 1997). Furthermore, there is need for the validation of the current serological tests for the pigs population in the country, as the prozone effect may interfere with the CFT assay results and also, it is imperative to ascertain the absence of antibodies to *Y. enterocolitica* which cross‐reacts with seropositivity for brucellosis as reported by the OIE (2016). The low seropositivity for porcine brucellosis (0.13%) detected in our study could be attributed to natural exposure, and as reported for the first time in the country, it is comparable to 0.25% reported in pigs in Uganda (Erume et al., 2016). However, both studies did not report on the elimination of *Yersinia enterocolitica* serotype O:9 as recommended by the OIE (2016) in the pigs, which is known to cross‐react with serological test results for porcine brucellosis. The isolation of *Brucella* from livestock (Caine et al., 2017; Kolo et al., 2019) and humans (Schrire, 1962; Wojno et al., 2016) in South Africa emphases the need to associate seropositivity for brucellosis with bacteriological isolation results.

Furthermore, it is impossible to ascertain the infecting *Brucella* species using serological tests, irrespective of the antigen (*B. melitensis* or *B. abortus*) or host species tested (Ariza, 1999; OIE, 2013a, 2013b; Spink, 1956). This is because of the dominance and overlapping nature of the C epitope of smooth brucellae (Alonso‐Urmeneta et al., 1998). This study emphasizes that bacteriological isolation is necessary to ascertain the infecting *Brucella* species and to understand the epidemiology when different host species are managed together or share grazing grounds and water sources. However, identification and typing of *Brucella* species by conventional procedures are difficult and molecular methods are preferred for typing strains once these are isolated as described (Kolo et al., 2019; OIE, 2013a).

Based on the risk factor evaluation, it will appear that the restructuring of the testing scheme in 2010, from the period of announcement that ‘*Only the accredited laboratories can test for brucellosis*’, the odds ratios for *Brucella* seropositivity in livestock have increased, particularly in the sheep and cattle, and in the year 2010, for goats too. However, no significant increase in odds of seropositive sample has been noticed in pigs (Table 1). Whether this is due to (a). Increasing volume of export that mandated more samples to be presented for testing, (b). Increasing awareness of testing that boost sample submission, (c). Better standardization since only accredited laboratories are now involved and (d). Sheer increase in numbers of truly seropositive samples due to increasing infection nationwide is unclear.

It is of diagnostic relevance that the vaccination status of all the animals serologically tested (RBT and CFT) for brucellosis in the diagnostic laboratory is unavailable. More so, the live attenuated *B. abortus* S19 vaccine used in the country has the potential to interfere with sero‐diagnosis (OIE, 2009). However, the cattle tested were suspected clinical cases of brucellosis, most likely due to natural exposure to virulent *Brucella* field strains. Cattle that have been vaccinated with *B. abortus* S19 or sheep with *B. melitensis* Rev 1 between 3 and 6 months are usually considered to be infected if the sera give positive fixation at a titre of 30 or greater ICFTU/ml when the animals are tested at an age of 18 months or older (OIE, 2013a). One way to avoid potential interference of vaccines in brucellosis sero‐surveillance of testing is the recommended use of the rough *B. abortus* RB51 vaccine (Sowa et al., 1992), which do not induce antibodies detected in routine testing for brucellosis. However, this vaccine is not supplied by the government and it is more expensive than *B. abortus* S19 vaccine but can be used for heifer or cow at any age. RB51 vaccine causing abortion has been reported but to a lesser extent than S19 (Palmer et al., 1996). The failure of diagnostic laboratories to capture information on potential risk factors such as age, sex, breed, status of the herd from where the animals originate limits the application of the data for the country and veterinary services. Such data are imperative for the control and eradication of brucellosis as documented by others (Asante et al., 2019; Idrissi, 2014). Countries that have successfully eliminated or controlled brucellosis in livestock have achieved this by instituting control measures based on the demographic records of brucellosis as reported by others (Maloney & Fraser, 2001; Smirnova et al., 2013). The acquisition of important information on risk factors in animals tested for brucellosis at veterinary laboratories in the country will be invaluable when used in conjunction with the seropositivity data.

The finding that Gauteng province had the highest seropositivity for brucellosis 47.90%(21,389/44,687) (Table S3) among all seropositive samples in the nine provinces may be attributed, in part, to the location of the Bacterial Serology Laboratory, ARC‐OVR in Pretoria (Gauteng province), which facilitates easy access and offers convenience to farmers and animal health workers to transport animal samples to the laboratory. This phenomenon was observed to be a factor in disease clustering in a study in California (Soberano et al., 2009). Within the Gauteng province, official annual report (2015–2016) shows a brucellosis seroprevalence of 1.27% in cattle population (GDARD, 2016), which is lower than the seropositivity of 4.06% recorded in this study in Gauteng (Figure 3). The difference in the findings of both studies may be explained in part by the duration of the studies (1 vs. 9 years) and the study design (prevalence study on general cattle population vs. seropositivity in suspect cases).

The disproportionate quantity of samples submitted for testing from Gauteng province (70.10%) is not reflective of the spatial distribution of livestock resources in South Africa. While the immediate reasons for this observation are unknown, the ARC‐OVR, the lead laboratory for brucellosis testing scheme is located in Gauteng. Spatial clustering of sample submission has been documented in other countries as influencing more sample submission from contiguous and proximity locations to the laboratories (Ekong et al., 2018; Soberano et al., 2009). A possible decentralization of sample collection and collation centres may partially correct this observed skewed sampling. Perhaps, the pulling of all results from other accredited laboratories earlier identified in the material and method section, may also reduce the margin of disparities observed in this study.

The analysis of the retrospective data in this study has demonstrated that the South African government's current funding of the brucellosis serological tests has a data with limited information from which only minimal inferences can be made to the general livestock population in the country. We acknowledge the many constrains encountered by veterinary services in South Africa and therefore suggest government implement a continuous reporting of disease surveillance and prevalence in annual reports from the central veterinary laboratory that can be easily collated. The recording of brucellosis data can be significantly improved by centralizing the data from veterinary laboratories to provide a better estimate of the brucellosis status in South Africa. If a centralized database is not possible, serological and bacteriological results can both be recorded in the annual report. These disease data can be used by government to identify disease problems and to optimize disease management and control efforts by using existing resources. This could improve communication of disease outbreaks despite limited resources experienced in government.

## 5 LIMITATIONS OF THIS STUDY

The limitations in this study include:

- Major inferences cannot be made because important variables (risk factors) such as the age, vaccination status, sex, breed and sources of the animals, were missing from the database.
- Serology can only be a presumptive test since other pathogens (e.g. *Y. enterocolitica*, O:9) can cross‐react with the tests, thereby leading to false‐positive results and;
- Sampling of the animals from the different provinces was biased because other than compulsory testing of dairy and stud cattle herds include mainly suspect cases of brucellosis that were tested, which may not be representative of cattle population in the country.
- Furthermore, the herd prevalence could not be determined based on the status of animals tested coupled with the lack of information on the herds of origin of the animals tested in the current study.

## 6 CONCLUSIONS

In conclusion, the review and analysis of 9‐year data on brucellosis from the Bacterial Serology Laboratory, ARC‐OVR has demonstrated that brucellosis in livestock is endemic in South Africa. Although the data lacked information on the vaccination status of the tested livestock, the fact that vaccinated animals are protected against brucellosis and thus will not exhibit clinical manifestation or require laboratory diagnosis, it is concluded that the seropositivity for the disease detected in our study reflects the natural exposure to *Brucella* spp. This study has provided baseline data that would lead to the improvement of informed policy to control the disease in the country. Although some vital information about the animals were missing, the information from our study can be used pro‐actively to drive, monitor, change or formulate policies to mitigate the challenges brought about by brucellosis in the livestock sector of the country. This study identified the primary focus on bovine brucellosis, lack of linking brucellosis serological and bacteriological results, lack of centralized database and as mentioned the lack of vaccination status as gaps in the recording of data in South Africa.

## 7 RECOMMENDATIONS

Considering that the data available at the Bacterial Serology Laboratory, ARC‐OVR are grossly inadequate for analysis and drawing inferences on the epidemiology and important variables associated with brucellosis in livestock in South Africa, it is important that all samples from the provincial laboratories to the Bacterial Serology Laboratory, ARC‐OVR should be accompanied by information such as animal sources, sex, age, breed and vaccination status of the animal.

To date in the country, vaccination of livestock against brucellosis with no stipulations on the types of vaccines allowed in the country, coupled by the fact that no compensation is paid to farmers of slaughtered brucellosis‐positive livestock which may discourage their willingness to report the disease to the authorities. This practice therefore poses public health significance to farm/abattoir workers and consumers. It is therefore recommended that there is a need for standardized and target national *Brucella* program as well as the provision of resources for vaccination and indemnity.

At present in the country, with regard to brucellosis, animal health professionals including veterinarians and animal health assistants, are not trained or lack experience in disease tracebacks and programme implementation. There is therefore the need to train these personnel in testing for diseases such as brucellosis, collecting appropriate samples from suspect animals and to be proficient in disease prevention and control implementation.

Finally, brucellosis is a reportable disease in South Africa and our study has identified gaps, such as the lack of invaluable information on the livestock tested and the testing of only suspect cases and export livestock have the potential to contribute to the under‐reporting of brucellosis in the country. It is therefore imperative to address these limitations to generate accurate data which will be essential for the development of an annual reporting and summary for program evaluation purposes.

## CONFLICT OF INTEREST

There is no conflict of interest with regards to this research.

## AUTHOR CONTRIBUTION

**Francis B. Kolo:** Conceptualization; Data curation; Formal analysis; Investigation; Methodology; Software; Validation; Visualization; Writing‐original draft; Writing‐review & editing. **Abiodun A. Adesiyun:** Conceptualization; Formal analysis; Funding acquisition; Methodology; Project administration; Resources; Supervision; Validation; Visualization; Writing‐review & editing. **Folorunso O. Fasina:** Conceptualization; Data curation; Formal analysis; Funding acquisition; Methodology; Resources; Software; Supervision; Validation; Visualization; Writing‐review & editing. **Andrew Pott:** Data curation; Formal analysis; Investigation; Methodology; Project administration; Software; Validation; Visualization; Writing‐review & editing. **Banenat B. Dogonyaro:** Data curation; Investigation; Methodology; Writing‐review & editing. **Charles T. Katsande:** Conceptualization; Data curation; Funding acquisition; Investigation; Methodology; Project administration; Resources; Validation; Writing‐review & editing. **Henriette van Heerden:** Conceptualization; Data curation; Formal analysis; Funding acquisition; Investigation; Methodology; Project administration; Resources; Supervision; Validation; Visualization; Writing‐review & editing.

## ETHICAL STATEMENT

Permission to perform the research was granted regarding Section 20 Animal Diseases Act, 1984 (Act number 34 of 1984), by the Department of Agriculture, Forestry and Fisheries, Reference number 12/11/1/1/6. Ethics approval was also granted by the University of Pretoria's Animal Ethics Committee, project number AEC12‐16.

### PEER REVIEW

The peer review history for this article is available at https://publons.com/publon/10.1002/vms3.363.

## Supporting information

Table S1‐S3
