# Supplementary material for: A retrospective study (2007–2015) on brucellosis seropositivity in livestock in South Africa
Source: Vet Med Sci. 2020 Oct 22;7(2):348–56. doi: 10.1002/vms3.363 (PMC8025618; doi:10.1002/vms3.363)
Supplement: Supplementary file 1 — Table S1‐S3 [file VMS3-7-348-s001.docx]

Table S1: Annual population of cattle, sheep and pigs in South Africa in nine provinces for the period 2017 to 2015

|  |  | Annual estimation of livestock population by year (DAFF, 2018): | | | | | | | | |
| --- | --- | --- | --- | --- | --- | --- | --- | --- | --- | --- |
| Province* | Livestocktype*^‡^* | 2007 | 2008 | 2009 | 2010 | 2011 | 2012 | 2013 | 2014 | 2015 |
| Western Cape | Cattle | 533,456 | 567,286 | 570,875 | 573,675 | 558,289 | 565,626 | 561,509 | 561,210 | 568,600 |
|  | Sheep | 2,667,370 | 2,719,800 | 2,748,108 | 2,716,349 | 2,715,819 | 2,770,569 | 2,768,044 | 2,845,399 | 2,816,500 |
|  | Goat | 242,798 | 230,965 | 229,921 | 224,536 | 226,880 | 224,693 | 225,300 | 222,087 | 216,600 |
|  | Pigs | 189,509 | 183,677 | 183,178 | 180,301 | 178,553 | 177,917 | 173,426 | 172,024 | 169,700 |
| Northern Cape | Cattle | 493,233 | 515,179 | 517,506 | 514,109 | 517,068 | 513,590 | 498,733 | 505,656 | 507,200 |
|  | Sheep | 6,398,526 | 6,204,217 | 6,214,617 | 6,119,201 | 6,054,733 | 6,018,088 | 5,983,536 | 5,917,349 | 6,015,000 |
|  | Goat | 525,169 | 526,146 | 528,454 | 527,444 | 532,699 | 522,925 | 513,955 | 511,537 | 506,500 |
|  | Pigs | 26,228 | 24,343 | 27,822 | 28,769 | 27,649 | 27,575 | 26,592 | 27,331 | 27,500 |
| Free State | Cattle | 2,359,137 | 2,300,764 | 2,392,894 | 2,252,289 | 2,348,420 | 2,338,998 | 2,283,810 | 2,306,706 | 2,312,000 |
|  | Sheep | 5,055,942 | 4,945,228 | 4,886,255 | 4,875,111 | 4,880,030 | 4,806,186 | 4,872,474 | 4,825,614 | 4,794,000 |
|  | Goat | 260,230 | 265,517 | 258,336 | 244,486 | 241,470 | 243,621 | 237,553 | 226,758 | 229,800 |
|  | Pigs | 133,852 | 129,254 | 132,537 | 129,948 | 125,258 | 125,824 | 126,161 | 122,829 | 120,000 |
| Eastern Cape | Cattle | 3,222,849 | 3,273,978 | 3,235,188 | 3,146,250 | 3,221,407 | 3,330,733 | 3,289,202 | 3,375,440 | 3,369,000 |
|  | Sheep | 7,501,575 | 7,571,170 | 7,589,870 | 7,316,381 | 7,302,429 | 7,294,003 | 7,156,560 | 7,025,292 | 7,114,000 |
|  | Goat | 2,520,608 | 2,428,176 | 2,385,218 | 2,355,392 | 2,328,371 | 2,358,504 | 2,292,306 | 2,269,179 | 2,244,000 |
|  | Pigs | 106,452 | 104,899 | 102,091 | 96,466 | 97,558 | 97,674 | 95,411 | 94,125 | 93,000 |
| KwaZulu-Natal | Cattle | 2,884,131 | 2,788,615 | 2,782,387 | 2,761,318 | 2,759,242 | 2,799,954 | 2,752,856 | 2,721,376 | 2,732,000 |
|  | Sheep | 760,963 | 761,278 | 788,469 | 770,528 | 759,752 | 762,448 | 751,933 | 733,995 | 737,500 |
|  | Goat | 860,498 | 855,380 | 865,427 | 831,857 | 822,476 | 827,479 | 823,317 | 811,534 | 814,500 |
|  | Pigs | 153,934 | 159,413 | 158,635 | 157,212 | 157,379 | 156,990 | 154,183 | 155,532 | 152,500 |
| Mpumalanga | Cattle | 1,483,630 | 1,475,700 | 1,460,622 | 1,489,887 | 1,478,476 | 1,465,385 | 1,429,529 | 1,462,262 | 1,421,500 |
|  | Sheep | 1,633,752 | 1,796,190 | 1,798,423 | 1,750,850 | 1,765,198 | 1,774,933 | 1,775,599 | 1,738,792 | 1,743,000 |
|  | Goat | 98,584 | 95,060 | 94,724 | 89,873 | 90,421 | 90,088 | 90,229 | 90,011 | 88,600 |
|  | Pigs | 143,640 | 129,392 | 123,534 | 128,379 | 128,767 | 126,831 | 124,272 | 123,924 | 124,000 |
| Limpopo | Cattle | 1,047,498 | 1,084,220 | 1,073,041 | 1,072,246 | 1,076,720 | 1,057,989 | 1,061,997 | 1,056,141 | 1,018,500 |
|  | Sheep | 245,523 | 250,572 | 266,646 | 257,653 | 262,362 | 258,996 | 258,149 | 257,720 | 250,800 |
|  | Goat | 1,075,634 | 1,310,895 | 1,313,255 | 1,276,543 | 1,230,461 | 1,143,393 | 1,125,454 | 1,055,032 | 1,074,000 |
|  | Pigs | 398,531 | 379,761 | 385,894 | 378,482 | 376,260 | 372,530 | 369,153 | 368,280 | 360,000 |
| Gauteng | Cattle | 263,385 | 269,863 | 260,574 | 258,838 | 260,671 | 260,809 | 257,603 | 255,160 | 252,000 |
|  | Sheep | 93,676 | 106,472 | 105,232 | 103,684 | 103,742 | 104,686 | 98,283 | 101,588 | 102,400 |
|  | Goat | 45,051 | 44,582 | 44,782 | 40,437 | 41,383 | 41,321 | 41,462 | 37,911 | 41,000 |
|  | Pigs | 181,574 | 182,704 | 182,889 | 178,806 | 180,322 | 181,075 | 177,763 | 182,474 | 168,500 |
| North West | Cattle | 1,814,303 | 1,760,050 | 1,799,677 | 1,762,010 | 1,799,887 | 1,764,993 | 1,696,693 | 1,707,438 | 1,702,000 |
|  | Sheep | 679,844 | 707,969 | 705,532 | 697,958 | 706,427 | 681,189 | 667,993 | 659,502 | 636,800 |
|  | Goat | 763,145 | 738,261 | 734,842 | 738,200 | 723,875 | 703,014 | 689,914 | 678,882 | 670,000 |
|  | Pigs | 336,028 | 330,165 | 327,424 | 321,703 | 320,004 | 315,919 | 319,694 | 315,504 | 322,000 |
| Total | **Cattle** | **14,101,622** | **14,035,655** | **14,092,764** | **13,830,622** | **14,020,180** | **14,098,077** | **13,831,932** | **13,951,389** | **13,882,800** |
|  | **Sheep** | **25,037,170** | **25,062,896** | **25,103,152** | **24,607,715** | **24,550,492** | **24,471,098** | **24,332,571** | **24,105,251** | **24,210,000** |
|  | **Goat** | **6,391,716** | **6,494,982** | **6,454,959** | **6,328,768** | **6,238,036** | **6,155,038** | **6,039,490** | **5,902,931** | **5,885,000** |
|  | **Pigs** | **1,669,749** | **1,623,608** | **1,624,004** | **1,600,066** | **1,591,750** | **1,582,335** | **1,566,655** | **1,562,023** | **1,537,200** |

*^‡^*The management of livestock is similar across the nine provinces: For cattle (feedlots, cow-calf and rural???), for sheep (feedlots and rural) and pigs (feedlot and rural)

**Table S2:** The number of animals tested, stratified by species and brucellosis seropositive results from 2007 to 2015

| Year | ^†^no. of samples tested^§^ | ^†^no. of Cattle  tested | Cattle positive ^‡^(%) | ^†^no. of Goats  tested | Goats positive ^‡^ (%) | ^†^no. of Sheep  tested | Sheep positive ^‡^ (%) | ^†^no. of Pigs  tested | Pigs positive ^‡^ (%) |
| --- | --- | --- | --- | --- | --- | --- | --- | --- | --- |
| 2007 | 76506 | 67441 | 2524  (3.74) | 3324 | 17 (0.51) | 5053 | 20 (0.39) | 688 | 2  (0.29) |
| 2008 | 83014 | 71348 | 3053  (4.28) | 4453 | 0  (0.00) | 6493 | 91 (1.40) | 720 | 0  (0.0) |
| 2009 | 95717 | 86802 | 3651  (4.21) | 3433 | 22  (0.64) | 4966 | 26 (0.52) | 516 | 0  (0.0) |
| 2010 | 85994 | 75862 | 6292  (8.29) | 3131 | 147  (4.69) | 6704 | 271 (4.04) | 297 | 0  (0.0) |
| 2011 | 77871 | 70517 | 5355 (7.59) | 4039 | 1  (0.02) | 2941 | 148 (5.03) | 374 | 1  (0.27) |
| 2012 | 71536 | 65680 | 4300 (6.55) | 2856 | 1  (0.03) | 2893 | 62 (2.14) | 107 | 0  (0.0) |
| 2013 | 87007 | 83100 | 5972 (7.19) | 2006 | 0  (0.00) | 1627 | 56 (3.44) | 274 | 1  (0.36) |
| 2014 | 90266 | 82740 | 7597 (9.18) | 3003 | 0  (0.00) | 4453 | 74 (1.66) | 70 | 0  (0.0) |
| 2015 | 96365 | 88049 | 4922 (5.59) | 3722 | 1  (0.03) | 4542 | 80 (1.76) | 52 | 0  (0.0) |
| TOTAL | **764,276** | **691539** | 43666 | **29,967** | 189 | **39,672** | 828 | **3,098** | **4** |

*§For the four species (cattle, sheep, goats and pigs), † = Number, ‡ = percentage*

**Table S3:** Seropositivity of brucellosis stratified by animal species and province, 2007 to 2015, number of animals tested with positives and stratified by positives from each species within the nine provinces from 2007 to 2015.

|  | Cattle | | Goats | | Sheep | | Pigs | | Overall | |
| --- | --- | --- | --- | --- | --- | --- | --- | --- | --- | --- |
| Province | **^†^no. tested** | **^†^no. ^‡^(%)**  **positive** | **^†^no. tested** | **^†^no. ^‡^(%) positive** | **^†^no. tested** | **^†^no. ^‡^(%) positive** | **^†^no. tested** | **^†^no. ^‡^(%) positive** | **^†^no. tested** | **^†^no. ^‡^(%) positive** |
| Eastern Cape | 9941 | 461 **(4.64)** | 3994 | 17  **(0.43)** | 8989 | 100  **(1.11)** | 3 | 0  **(0.0)** | 22927 | 578  **(2.52)** |
| Gauteng | 523115 | 21228 **(4.06)** | 6131 | 146  **(2.38)** | 3587 | 11  **(0.31)** | 2929 | 4  **(0.14)** | 535762 | 21389  **(3.99)** |
| KwaZulu Natal | 8025 | 185 **(2.31)** | 2658 | 21  **(0.79)** | 477 | 15  **(3.14)** | 79 | 0  **(0.0)** | 11239 | 221  **(1.97)** |
| Limpopo | 44151 | **8684**  **(19.67)** | 2998 | 0  **(0.00)** | 2099 | 11  **(0.52)** | 0 | 0  **(0.0)** | 49248 | 8695  **(17.65)** |
| Mpumalanga | 20928 | 1793  **(8.57)** | 474 | 0  **(0.00)** | 827 | 13  **(1.57)** | 38 | 0  **(0.0)** | 22267 | 1806  **(8.11)** |
| Northern Cape | 18869 | 4985 **(26.42)** | 3050 | 0  **(0.0)** | 9301 | 274  **(2.94)** | 0 | 0  **(0.0)** | 31220 | 5259  **(16.8)** |
| North West | 45155 | 3947 **(8.74)** | 8200 | 4  **(0.05)** | 4700 | 46  **(0.98)** | 45 | 0  **(0.0)** | 58099 | 3997  **(6.88)** |
| Free State | 17138 | 2312 **(13.50)** | 2172 | 1  **(0.05)** | 9258 | 338  **(3.65)** | 4 | 0  **(0.0)** | 28572 | 2651  **(9.28)** |
| Western Cape | 4217 | 71 **(1.68)** | 290 | 0  **(0.0)** | 434 | 20  **(4.61)** | 0 | 0  **(0.0)** | 4941 | 91  **(1.84)** |
| TOTAL | **691539** | **43666**  **(6.31)**  **(^§^CI:6.26-6.37)** | **29967** | **189**  **(0.63)**  **(^§^CI:0.55-0.73)** | **39672** | **828**  **(2.09)**  **(^§^CI:1.95-2.23)** | **3098** | **4**  **(0.13)**  **(^§^CI:0.05-0.33)** | **764276** | **44687**  **(5.85)**  **(^§^CI:5.79-5.90)** |

*† = Number, ‡ = percentage,* ***§ =*** *Confidence Interval = 95%*
